# Erratum

**Published:** 2013-04-19

## Vol. 62, No. 13

An error occurred in the first sentence of the announcement, “World Health Day — April 7, 2013,” on page 237. The sentence should read as follows: “World Health Day and the **65th** anniversary of the World Health Organization (WHO) will be observed April 7.”

